# Emerging pandemics: Lesson for one‐health approach

**DOI:** 10.1002/vms3.361

**Published:** 2020-10-08

**Authors:** Krishna P. Acharya, Supram H. Subramanya, Dinesh Neupane

**Affiliations:** ^1^ Animal Quarantine Office, Department of Livestock Services Kathmandu Nepal; ^2^ Manipal College of Medical Sciences Pokhara Nepal; ^3^ Johns Hopkins Bloomberg School of Public Health Baltimore MD USA

Historically, there have been a number of significant infectious disease outbreaks. For example Bubonic Plague of 1665, Spanish flu of 1918, SARS‐CoV of 2002–2003, H_1_N_1_ influenza of 2009, Zika pandemics of 2013 and the ongoing COVID‐19 pandemic. These major outbreaks have emerged from animals; Bubonic plague from rat, Spanish flu from birds (CDC, 2018; Taubenberger & Morens, 2006), spread of SARS from bats to civets to humans(Guan et al., 2003), spread of MERS‐CoV from vespertilionid bats to camelids to humans (Corman et al., 2014) and the close relatedness of SARS‐CoV‐2 to bat and pangolin viruses (Lau et al., 2020; Wu et al., 2020; Zhou et al., 2020). Over 30 new human pathogens have emerged in the last three decades, 75% of which have originated in animals (Jones et al., 2008). Animal borne emerging infectious diseases can cause significant economic shocks.

More than 75% of new or emerging infectious diseases spreads from animals to humans (anthropozoonoses) through various routes (FAO & USAID, 2019; Munyua et al., 2019). Interestingly, some of the common pathogens of humans such as *Mycobacterium tuberculosis, Methicillin‐resistant Staphylococcus aureus (MRSA), Influenza A virus, Cryptosporidium parvum, Ascaris lumbricoides* can be transmitted from humans to animal populations (zooanthroponoses) (Messenger et al., 2014). The transmission of pathogens between animals and humans requires target control measures/interventions (Ajit, 2019). The close relationship between animals and humans can increase the risk for transmission of drug‐resistant infections. Due to the intimate human animal‐environmental interface, controlling these epidemics/pandemics requires multi‐sectoral collaboration between numerous disciplines, which suggests a One‐Health approach to understand the biological, social, environmental and genetic determinants of health and diseases of humans and animals (Mackenzie & Jeggo, 2019). To prevent and control these emerging diseases, we may need to develop more advanced systems such as genomic precision surveillance platforms that help to detect and report the cases of outbreaks on time (Acharya et al., 2019).

Additionally, enhanced public health infrastructure, population control, sanitation, strict food safety measures, scrutinized international travel and trade are anticipated (Munyua et al., 2019). Besides, environmental factors like evolving patterns of land and water use, the despondency of habitats, intensive farming, pesticide use, wildlife trade and emission of the atmosphere's greenhouse gases need to be properly measured and regulated in harmony with the nature. For complex monitoring and policy interventions, an inter‐sectoral health system collaboration is required. In particular, communication at regional, national and international levels across the one‐health sectors to limit the impact of these changes on the health of all (FAO & USAID, 2019). The large scale implementation of the One‐Health approach is limited; a few projects have been effectively implemented in outbreaks, for example, to prevent river blindness (Onchocerciasis) (Cantey et al., 2018; Voegele & Kemper, 2018) in Africa and Avian Influenza in Asia (The World Bank, 2011) and Africa (Dixit, 2015).

The One‐Health approach can be highly efficient in terms of resource‐use efficiency. An analysis by the World Bank suggested strengthening human and animal health capacity by the One‐Health approach could result in 10%–30% cost saving in surveillance and communication cost (World Bank, 2012). A study by Rostal *et al*., in sub‐Saharan Africa, on the prevalence of Rift Valley Fever (RVF), demonstrated that the One‐Health surveillance system was effective in detecting cases of RVF. It was concluded that these cases would have likely been missed with traditional surveillance systems (Rostal et al., 2018). Economic analysis of this risk reduction and early detection, demonstrated a cost saving of 35% (Rostal et al., 2018). Qatar has successfully combated MERS‐CoV through active surveillance, inter‐sectoral collaboration and joint investigation through the One‐Health approach (Farag et al., 2019). A similar study using a One‐Health approach with an integrated human and animal disease laboratory in Winnipeg, Canada demonstrated cost savings of 26% per year (World Bank, 2012). Thus, implementation of One‐Health approach carries a potential for the prevention and control of trans‐boundary zoonotic diseases, such as Avian Influenza, Swine Influenza, SARS, MERS, Q‐fever and COVID‐19 efficiently and cost‐effectively. Often, the public health authority, however, remiss the significance of animal and environmental health sectors, as a result, it has been challenging to control such pandemics. Till now, holistic systems of integrating human health, animal health and environment‐related disciplines have not been implemented. Thus, we are failing to understand the pivotal role of animal and environmental health sectors to prioritize the “One‐Health” approach.

It is essential to prepare all the countries for possible future pandemics by appropriate allocations of financial resources, international collaboration, strengthening the veterinary services and public health systems. That needs a financing mechanism to disburse the required funds for a rapid and effective response to pandemics (Voegele & Kemper, 2018). Besides financial resources, human resource investments are required such as in field‐based healthcare workers, epidemiologists, microbiologists, infection prevention scientists and control specialists and veterinarians. The workforce should be well trained, adequately equipped and an ability to rapidly deploy. Additionally, timely communication of real‐time epidemiological data is essential.

The ongoing COVID‐19 pandemic has also delivered a clear memorandum for early detection and isolation of infected cases and enforcing measures to halt further transmissions (travel restriction, social distancing, quarantine and hygiene) to diminish the risk of infections. For example few countries like New Zealand has controlled COVID‐19 by rapid science‐based risk assessment, early countrywide locked down, science‐based advocacy and implementation of strict intervention measures such as border control, community‐transmission control measures and case‐based control measures (Po‐Chang et al., 2020). In parallel, Taiwan has reduced the cases of COVID‐19 by availability of free rapid testing centres and mass screening, advanced early warning systems and encouraging wearing face masks (Christina & Michelle, 2020). Additionally, timely notification of outbreaks to the international community and transparency during a health emergency is crucial to halt the global spread of the disease. Early detection, control and containment of new threats through a holistic ‘One‐Health’ approach are, thus, vital in decreasing morbidity and mortality, and to reduce public health, and economic impacts of animal‐associated pandemics. However, challenges, especially in low‐ and middle‐income countries (LMICs), remain concerning, resources for veterinary laboratory diagnosis and resources to implement more comprehensive control and prevention measures. The outbreak by a novel infectious agent can emerge at any time in any country and spread globally. A regional and global implementation and integration of the One‐Health approach is fundamental for future global health security. Thus, it is essential to work together to implement a One‐Health strategy to address complex infectious disease health challenges.

## DATA SHARING STATEMENT

Data sharing is not applicable to this article as no new data were created or analysed in this study.

## ETHICS STATEMENT

The authors confirm that the ethical policies of the journal, as noted on the journal's author guidelines page, have been adhered to. No ethical approval was required as this is a letter with no original research data.

## CONFLICT OF INTEREST

The authors declare that there is no conflict of interest.

## AUTHOR CONTRIBUTION

**Krishna Prasad Acharya:** Conceptualization; Resources; Writing‐original draft; Writing‐review & editing. **Supram Hosuru Subramanya:** Conceptualization; Resources; Writing‐original draft; Writing‐review & editing. **Dinesh Neupane:** Resources; Validation; Writing‐review & editing.

### PEER REVIEW

The peer review history for this article is available at https://publons.com/publon/10.1002/vms3.361.
